# Risk of non-reproductive cancer in men from couples conceiving through assisted reproduction: a Swedish nation-based register study

**DOI:** 10.1007/s10654-026-01368-4

**Published:** 2026-02-21

**Authors:** Peter Zarén, Michael Kitlinski, Aleksander Giwercman, Yvonne Lundberg Giwercman, Angel Elenkov

**Affiliations:** 1 Department of Translational Medicine, Lund University, Malmö, Sweden; 2 Reproductive Medicine Centre, Skåne University Hospital, Östra Varvsgatan 11 F, Plan 5, Reproduktionsmedicinskt centrum, Malmö, 20502 Sweden; 3 Faculty of Medicine, Medical University of Gdansk, Gdańsk, Poland

**Keywords:** Male infertility, Cancer, Assisted reproduction

## Abstract

Whereas the link between impaired male fertility and risk of testicular and prostate cancer is well established, studies investigating non-reproductive cancer risk among men with impaired reproductive function are scarce and show conflicting results. The aim of the study was to compare the risk of developing non-reproductive cancers in men achieving paternity through assisted reproduction as compared to those conceiving naturally. All first-time fathers *n* = 1 137 829 in Sweden during the period January 1994 to December 2014 were included and followed from the time of conception until date of cancer diagnosis, death, or end of follow-up (31st of December 2014). Conception by intracytoplasmic sperm injection (ICSI) or use of donated spermatozoa was used as proxy for impaired male fertility. As controls we used males who conceived spontaneously. National register based cohort study. By linking the Swedish multigeneration register with the Swedish medical birth register (MBR), all men conceiving their 1st child between 1994 and 2014 in Sweden were identified (*n* = 1 181 490). Information on mode of conception was derived from the MBR or the Q-IVF register. Men with history of cancer prior to conception, missing covariate data, and those with missing information to estimate gestational age were excluded (*n* = 43 661). From the Swedish cancer registry data was retrieved on cancer diagnoses during follow-up. Fathers´ fertility status was based on mode of conception i.e. naturally, standard in-vitro fertilization (IVF) or ICSI. Diagnosis of any type of non-reproductive cancer according to International Classification of Diseases 7th revision (ICD-7) after the time of conception. Among 1 137 829 men, 20 142 and 14 540 achieved paternity through IVF and ICSI treatment, respectively. ICSI-fathers and those using donated spermatozoa were, as compared to those conceiving spontanouesly, at an increased risk of non-reproductive cancers (adjusted hazardo ratio (aHR) 1.3; 95%CI 1.1–1.5), foremost colon (aHR 1.7; 95%CI 1.1–2.7), rectum (aHR 1.8. 95%CI 1.1-3.0) or thyroid gland cancer (aHR 3.3; 95%CI 1.7–6.2). Men achieving paternity after ICSI treatment or by using donated spermatozoa, in other words those with most severely hampered fertility, were at increased risk of colorectal and thyroid cancer. For this subpopulation of men, an extended health check-up at the time of fertility treatment as well as at long-term follow-up, should be considered.

**Supplementary Information:**

The online version contains supplementary material available at 10.1007/s10654-026-01368-4.

## Introduction

According to a recent report from the *World Health Organization*, it is estimated that 1 in 6 couples will suffer from infertility at some point in their life [1]. In half of these cases, the underlying cause of the inability to conceive a child spontaneously is attributed to impaired male fertility [2]. Although many subfertile men may still be able to achieve paternity through use of powerful assisted reproduction techniques (ARTs), such as in-vitro fertilization (IVF) or intracytoplasmic sperm injection (ICSI), newly accumulated evidence suggest that men with impaired fertility are prone to long-term adverse health outcomes, including an increased risk of some types of urological cancers, as well as metabolic and cardiovascular diseases [3–7]. Moreover, subfertile men are at an increased risk of early death [8]. As such, impairment of fertility has been suggested as a general marker for health in men, yet no clinical follow-up nor screening program exists for this category of men.

A substantial amount of data has in particular linked male subfertility with an increased risk of testicular cancer [9]. The risk of prostate cancer has also been investigated in this context, showing that men conceiving by ICSI, which in Sweden is primarily used for male factor subfertility [10], were at an increased risk of developing prostate cancer with earlier onset compared to men conceiving naturally [4]. An increased risk of prostate cancer-related death among childless men, has also been seen in other cohorts [11, 12]. However, regarding the risk of developing malignancies other than those of the male reproductive tract, available data is scarce and often conflicting [13–16]. Genetic alterations, potentially increasing risk of both male infertility and cancer, have been demonstrated, e.g. mutations or downregulation of mismatch repair genes MLH1 and MSH2 [17–20]. However, it is unclear whether this, on a population level, translates into an increased risk of non-reproductive cancer among infertile men.

Thus, using Swedish nationwide registers with decades of follow-up time we aimed to investigate the risk of non-reproductive cancer among men who achieved paternity by ICSI or using donated spermatozoa - both being proxy for male subfertility - as compared to men who conceived naturally.

## Methods

### Data extraction from population-based registers

The Medical Birth Register (MBR) contains data on all children born in Sweden since 1973. By linking the MBR to the Swedish multi-generation register, which contains information on the identity of the parents to each person living in Sweden, we identified all fathers to children born between 1994 and 2014 (*n* = 1 181 490). For each of these men, his first child born during the study period was considered. The mode of conception for each child was obtained from the MBR (for children born before 2007) and from the Swedish National Quality Registry for Assisted Reproduction (for children born 2007 and onwards). Using information from the MBR on gestational length, the date of conception was estimated for each child. Information on any cancer diagnoses for the fathers were obtained by linking to the Swedish Cancer Register. Since cancer treatment may have a negative impact on fertility, fathers with a history of cancer prior to conception (*n* = 5295) as well as fathers for whom the child’s date of conception could not be estimated (*n* = 871) were excluded, along with 37 495 of cases with missing covariate data, leaving 1 137 829 fathers in the final study population.

Cancer diagnoses were categorized according to their first three digits in the International Classification of Diseases 7th revision (ICD-7), available for all diagnoses in the Swedish Cancer Register during the entire study period, with the exception of lymphatic and hematopoietic cancers which were registered according to ICD-8. Non-reproductive cancer was defined as diagnosis codes 140–209, excluding 177–179 which represents cancers of the prostate, testis, and other/unspecified male genital organs.

The Regional Ethical Board in Lund, Sweden, approved the study (No 2015/670).

## Statistical analyses

The risk of non-reproductive cancer was analyzed with respect to mode of conception (natural conception, standard IVF, or ICSI/donated sperm) using Cox regression. Men achieving paternity through use of ART with donated sperm were grouped together with ICSI, as both are indicative of more pronounced male subfertility. The underlying time scale was from date of conception for the fathers first child until date of cancer diagnosis, death, or end of follow-up (31st of December 2014). Men were followed from the date of conception of their child until 31 st December 2014. The follow-up time for men ranged from 172 to 7947 days, with a median of 4499 days. The uneven follow-up times for the men were handled by analyzing the data using Cox Regression models. These models work by comparing rates of cancer diagnosis events over time, rather than the number of events over a fixed observation time, and therefore automatically handle uneven observation times among the men. Death was considered a censoring event in both cohorts. Adjustment was performed for age, educational level and date of conception. Educational level was defined according to categories 0–8 using the International Standard Classification of Education 2011 [21] at time of child birth.

To test if an increased risk of non-reproductive cancer could be due to an increase in reproductive cancer presenting as cancer of unknown primary (i.e. as primary manifestation of metastatic disease), a post-hoc sensitivity analysis was performed where diagnoses possibly representing cancers of unknown primary were excluded from the definition of non-reproductive cancer. The ICD-7 cancer diagnoses excluded were: 156 (liver, secondary and unspecified), 163 (lung, unspecified as to whether primary or secondary), 165 (thoracic organs, secondary), 198 (lymph nodes, secondary and unspecified) and 199 (other and unspecified sites).

Continuous variables (age and date of conception) were adjusted for in the regression models using restricted cubic splines with four knots, with the exception of models analyzing individual cancer diagnoses which were adjusted linearly for age to avoid overfitting. Restricted cubic splines makes it possible to account for almost any relationship, both linear and non-linear, between the covariates and the dependent variable [22].

Covariates in Cox regression models were tested for non-proportionality using Grambsch and Thernau’s test for non-proportionality cox.zph in the R survival package [23]. When evidence of non-proportionality was found, the affected covariates were kept in the original models. However, these model violations were further explored using Cox models stratified on time for the affected covariates, as sensitivity analyses. This allowed for different covariate coefficients to be estimated for the early and late parts of follow-up, with the two time strata used being before and after half the median follow-up time.

P-values < 0.05 were considered statistically significant. All statistical analyses were conducted using R version 4.4.0 with the addition of packages ‘rms’ version 6.8-0.8.8.8 and ‘survival’ version 3.5–8.5 [23–25].

## Results

### Study population

A total of 1 137 829 fathers were included from the MBR, of which 20 142 conceived through standard IVF, 14 540 used ICSI/donated sperm (of which 355 used ART with donated sperm) and 1 103 147 conceived naturally (controls). The median age at conception was similar among men undergoing standard IVF and ICSI/donated sperm procedures (both 35 years), whereas men with natural conception were younger (median 31 years). The corresponding age ranges were 20–62 years, 21–57 years, and 15–81 years, respectively. Educational level at the time of childbirth, classified according to the International Standard Classification of Education 2011 (ISCED; categories 0–8), was comparable across all exposure groups, with a median level of 3 and a range of 1–8 in each group. There were no missing values for paternal age and educational level.

## Risk of non-reproductive cancer

Number of incident cases of non-reproductive cancer, adjusted and unadjusted hazard ratios (HR_adj_; HR) along with 95% confidence intervals (CI) according to mode of conception are shown in Table 1. Fathers conceiving using ICSI/donated sperm had a significantly increased risk for developing non-reproductive cancer with HR_adj_ 1.3 (95% CI 1.1–1.5), whereas no such increase was seen for fathers using IVF having HR_adj_ 0.98 (95% CI 0.86–1.1). Evidence for non-proportionality were found for the covariates paternal age and date of conception. Models where these covariates were time-stratified, performed as a sensitivity analysis, showed identical results.” Among the 355 men who became fathers using donated sperm, 2 (0.6%) developed non-reproductive cancer.

**Table 1:** Risk of non-reproductive cancer according to mode of offspring conception. Hazard ratios presented according to conception method, with natural conception set as reference group

	IVF	ICSI/donated sperm	Natural conception
Non-reproductive cancer			
Number	232	192	12,950
HR_adj (95% CI)	0.98 (0.86–1.1)	1.3 (1.1–1.5)	ref
P-value	0.71	0.00026	ref
HR (95% CI)	1.3 (1.2–1.5)	2 (1.7–2.2)	ref
P-value	< 0.001	< 0.0001	ref

*HR*_*adj*_ Adjusted hazards ratio, *HR* Unadjusted hazards ratio, *CI* Confidence interval, *IVF* Standard in vitro fertilization, *ICSI* Intracytoplasmic sperm injection

**Fig. 1:**
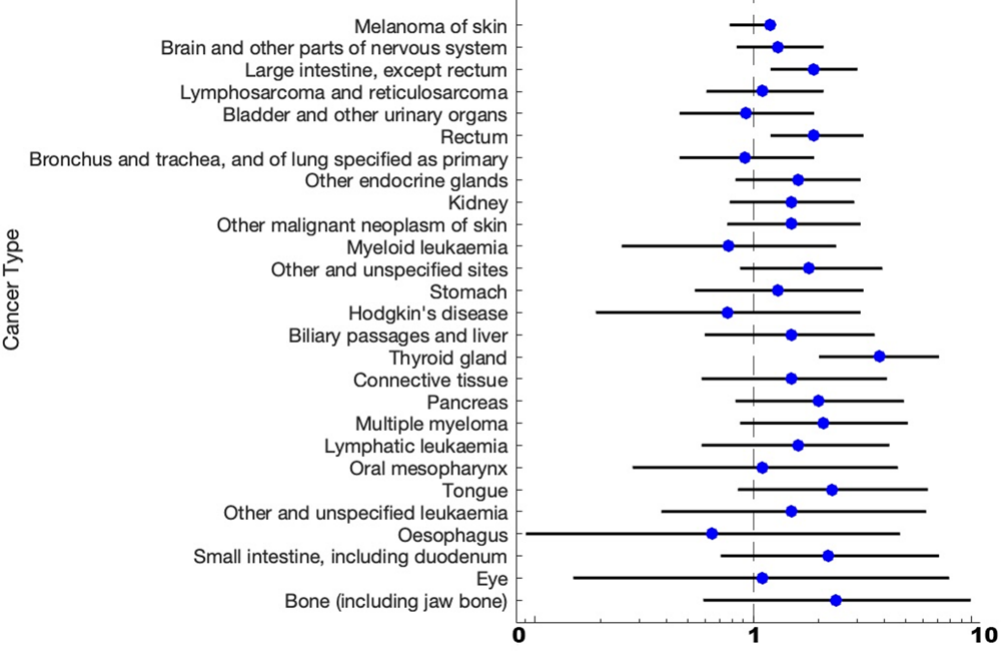
Forest plot displaying odds ratios (ORs) and 95% confidence intervals (CIs) for the risk of various cancer types in men treated with intracytoplasmic sperm injection (ICSI), compared to a control population

**Fig. 2:**
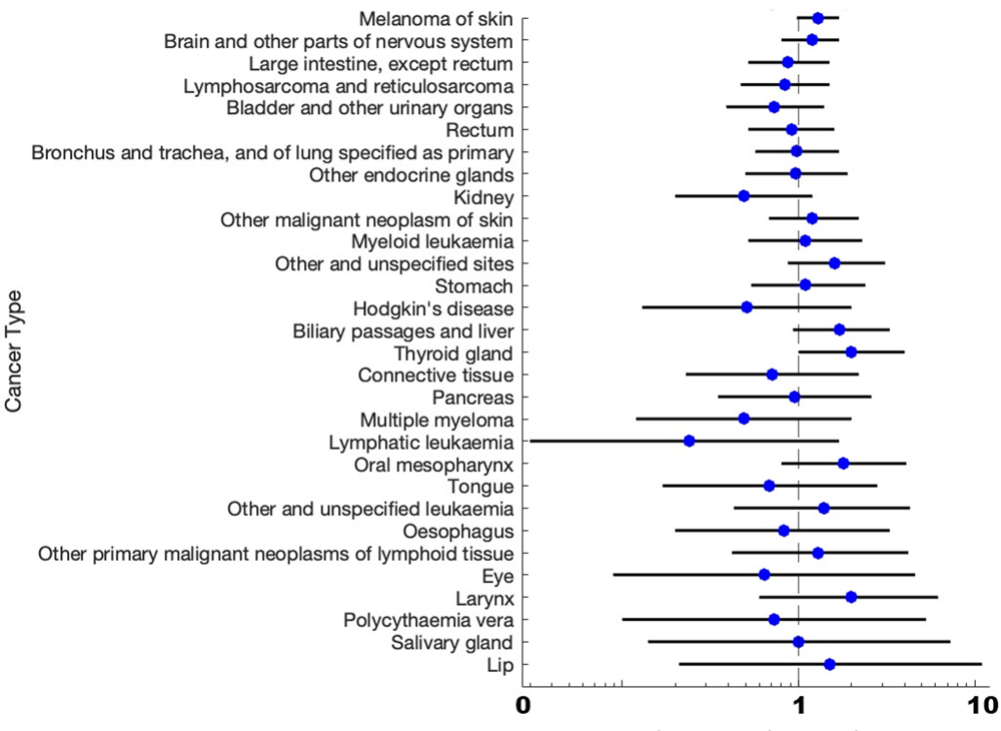
Forest plot displaying odds ratios (ORs) and 95% confidence intervals (CIs) for the risk of various cancer types in men treated with in vitro fertilization (IVF), compared to a control population

For subgroups of cancers, results are shown in Table 2. Men achieving paternity through ICSI/donated sperm as compared to those conceiving spontaneously, had a statistically increased risk of cancers in the large intestine, rectum and thyroid gland. In these analyses, the risk for cancer of the large intestine was HR_adj_ 1.7 (95% CI 1.1–2.7) and HR_adj_ 1.8 (95% CI 1.1–3.0.1.0) for rectal cancer. Risk of developing thyroid cancer in fathers conceiving through ICSI/donated sperm treatment was HR_adj_ 3.8 (95% CI 2.0–7.1.0.1) Performance of a post-hoc analysis of these cancer types, adjusting for all other covariates used in the analysis of non-reproductive cancer, showed similar estimates, giving HR_adj_ 1.7 (95% CI 1.1–2.7) for cancer of the large intestine, HR_adj_ 1.8 (95% CI 1.1–3.0.1.0) for rectal cancer and HR_adj_ 3.3 (95% CI 1.7–6.2) for thyroid cancer (Fig. 1). There was evidence for non-proportionality in all these Cox regressions, but the statistical significance remained in the time-stratified versions of these models. Non-proportionality was not possible to test in the model regarding thyroid cancer, due to a low number of cases. Noatbly, although not reaching the level of statistical significance in 21 of 33 remaining subgroups of cancer among ICSI fathers, the HR_adj_ was above 1.0. Results for men treated with IVF are presented in Fig. 2.

**Table 2:** Risk of site-specific cancers in men fathering through ICSI as compared to those achieving paternity naturally. Hazard ratios presented according to conception method, with natural conception set as reference

ICD-7	Cancer type	IVF	ICSI/donated sperm	Natural conception (control group)
190	Melanoma of skin			
	Number	47	25	2193
	HR_adj (95% CI)	1.3 (0.98–1.7)	1.2 (0.78–1.7)	ref
	P-value	0.068	0.46	ref
	HR (95% CI)	1.6 (1.2–2.1)	1.4 (0.95–2.1)	ref
	P-value	0.0021	0.086	ref
193	Brain and other parts of nervous system			
	Number	26	18	1323
	HR_adj (95% CI)	1.2 (0.8–1.7)	1.3 (0.84–2.1)	ref
	P-value	0.4	0.23	Ref
	HR (95% CI)	1.4 (0.94–2.94)	1.6 (0.99–2.5)	ref
	P-value	0.1	0.057	ref
153	Large intestine, except rectum			
	Number	15	19	931
	HR_adj (95% CI)	0.87 (0.52–1.5)	1.9 (1.2–3.2)	ref
	P-value	0.61	0.0066	ref
	HR (95% CI)	1.2 (0.74–2.74)	2.8 (1.8–4.4)	ref
	P-value	0.44	< 0.0001	ref
200	Lymphosarcoma and reticulosarcoma			
	Number	12	10	777
	HR_adj (95% CI)	0.84 (0.47–1.5)	1.1 (0.61–2.1)	ref
	P-value	0.55	0.68	ref
	HR (95% CI)	1.1 (0.64–2.64)	1.8 (1–3.3.3)	ref
	P-value	0.66	0.045	ref
181	Bladder and other urinary organs			
	Number	10	8	705
	HR_adj (95% CI)	0.73 (0.39–1.4)	0.93 (0.46–1.9)	ref
	P-value	0.32	0.85	ref
	HR (95% CI)	1.1 (0.58–2.58)	1.7 (0.88–3.3)	-
	P-value	0.8	0.11	-
154	Rectum			
	Number	12	15	690
	HR_adj (95% CI)	0.92 (0.52–1.6)	1.9 (1.2–3.2)	-
	P-value	0.78	0.012	-
	HR (95% CI)	1.4 (0.79–2.5)	7.4 (5.3–10)	-
	P-value	0.25	< 0.0001	-
162	Bronchus and trachea, and of lung specified as primary			
	Number	13	8	673
	HR_adj (95% CI)	0.98 (0.57–1.7)	0.92 (0.46–1.9)	-
	P-value	0.94	0.82	-
	HR (95% CI)	1.5 (0.87–2.6)	1.7 (0.84–3.4)	-
	P-value	0.15	0.14	-
195	Other endocrine glands			
	Number	9	9	557
	HR_adj (95% CI)	0.97 (0.5–1.9)	1.6 (0.83–3.1)	-
	P-value	0.92	0.16	-
	HR (95% CI)	1.2 (0.61–2.3)	2 (1.1–3.9)	-
	P-value	0.64	0.032	-
180	Kidney			
	Number	5	9	551
	HR_adj (95% CI)	0.49 (0.2–1.2)	1.5 (0.78–2.9)	-
	P-value	0.11	0.22	-
	HR (95% CI)	0.7 (0.29–1.7)	2.3 (1.2–4.4)	-
	P-value	0.43	0.014	-
191	Other malignant neoplasm of skin			
	Number	11	8	476
	HR_adj (95% CI)	1.2 (0.68–2.2)	1.5 (0.76–3.1)	-
	P-value	0.49	0.23	-
	HR (95% CI)	1.8 (1–3.3.3)	2.5 (1.2–5.2)	-
	P-value	0.048	0.0098	-
205	Myeloid leukaemia			
	Number	7	3	347
	HR_adj (95% CI)	1.1 (0.52–2.3)	0.77 (0.25–2.4)	-
	P-value	0.79	0.66	-
	HR (95% CI)	1.6 (0.76–3.2)	1 (0.33–3.2)	-
	P-value	0.23	0.95	-
199	Other and unspecified sites			
	Number	10	7	314
	HR_adj (95% CI)	1.6 (0.87–3.1)	1.8 (0.87–3.9)	-
	P-value	0.12	0.11	-
	HR (95% CI)	2.4 (1.3–4.5)	2.9 (1.4–6.1)	-
	P-value	0.0072	0.005	-
151	Stomach			
	Number	7	5	313
	HR_adj (95% CI)	1.1 (0.54–2.4)	1.3 (0.54–3.2)	-
	P-value	0.72	0.55	-
	HR (95% CI)	1.7 (0.8–3.6)	2.2 (0.92–5.2)	-
	P-value	0.17	0.077	-
201	Hodgkin’s disease			
	Number	2	2	320
	HR_adj (95% CI)	0.51 (0.13–2.13)	0.76 (0.19–3.1)	-
	P-value	0.34	0.71	-
	HR (95% CI)	0.42 (0.11–1.6)	0.63 (0.16–2.5)	-
	P-value	0.21	0.51	-
155	Biliary passages and liver			
	Number	10	5	301
	HR_adj (95% CI)	1.7 (0.93–3.3)	1.5 (0.6–3.6)	-
	P-value	0.083	0.4	-
	HR (95% CI)	2.6 (1.4–4.9)	2.4 (1–5.9.9)	-
	P-value	0.0026	0.05	-
194	Thyroid gland			
	Number	9	10	292
	HR_adj (95% CI)	2 (1–4)	3.8 (2–7.1.1)	-
	P-value	0.036	4.3e-05	-
	HR (95% CI)	2.2 (1.2–4.3)	4.1 (2.2–7.7)	-
	P-value	0.016	< 0.0001	-
197	Connective tissue			
	Number	3	4	248
	HR_adj (95% CI)	0.71 (0.23–2.2)	1.5 (0.58–4.1)	-
	P-value	0.55	0.39	-
	HR (95% CI)	0.84 (0.27–2.6)	1.9 (0.69–4.9)	-
	P-value	0.76	0.22	-
157	Pancreas			
	Number	4	5	226
	HR_adj (95% CI)	0.95 (0.35–2.6)	2 (0.83–4.9)	-
	P-value	0.92	0.12	-
	HR (95% CI)	1.4 (0.54–3.9)	3.4 (1.4–8.2)	-
	P-value	0.47	0.0071	-
203	Multiple myeloma			
	Number	2	5	223
	HR_adj (95% CI)	0.49 (0.12–2.12)	2.1 (0.87–5.1)	-
	P-value	0.32	0.1	-
	HR (95% CI)	0.72 (0.18–2.9)	3.3 (1.3–7.9)	-
	P-value	0.64	0.0091	-
204	Lymphatic leukaemia			
	Number	1	4	222
	HR_adj (95% CI)	0.24 (0.033–1.7.033.7)	1.6 (0.58–4.2)	-
	P-value	0.15	0.39	-
	HR (95% CI)	0.33 (0.047–2.4.047.4)	2.3 (0.85–6.2)	-
	P-value	0.27	0.1	-
145	Oral mesopharynx			
	Number	6	2	197
	HR_adj (95% CI)	1.8 (0.8–4.1)	1.1 (0.28–4.6)	-
	P-value	0.15	0.85	-
	HR (95% CI)	2.6 (1.1–5.8)	1.7 (0.42–6.8)	-
	P-value	0.023	0.46	-
141	Tongue			
	Number	2	4	161
	HR_adj (95% CI)	0.68 (0.17–2.8)	2.3 (0.85–6.3)	-
	P-value	0.59	0.099	-
	HR (95% CI)	0.94 (0.23–3.8)	3.4 (1.2–9.1)	-
	P-value	0.94	0.017	-
207	Other and unspecified leukaemia			
	Number	3	2	128
	HR_adj (95% CI)	1.4 (0.43–4.3)	1.5 (0.38–6.2)	-
	P-value	0.61	0.55	-
	HR (95% CI)	1.8 (0.57–5.6)	2.1 (0.54–8.5)	-
	P-value	0.32	0.27	-
150	Oesophagus			
	Number	2	1	124
	HR_adj (95% CI)	0.83 (0.2–3.3)	0.65 (0.091–4.7.091.7)	-
	P-value	0.79	0.67	-
	HR (95% CI)	1.3 (0.32–5.1)	1.1 (0.16–8.2)	-
	P-value	0.72	0.89	-
202	Other primary malignant neoplasms of lymphoid tissue			
	Number	3	0	119
	HR_adj (95% CI)	1.3 (0.42–4.2)		-
	P-value	0.62	NC	-
	HR (95% CI)	1.9 (0.6–5.9)		-
	P-value	0.28	NC	-
152	Small intestine, including duodenum			
	Number	0	3	111
	HR_adj (95% CI)		2.2 (0.71–7.1)	-
	P-value	NC	0.17	-
	HR (95% CI)		3.4 (1.1–11)	-
	P-value	NC	0.036	-
192	Eye			
	Number	1	1	89
	HR_adj (95% CI)	0.64 (0.089–4.6.089.6)	1.1 (0.15–7.9)	-
	P-value	0.66	0.93	-
	HR (95% CI)	0.85 (0.12–6.12)	1.6 (0.24–11.24)	-
	P-value	0.87	0.63	-
196	Bone (including jaw bone)			
	Number	0	2	85
	HR_adj (95% CI)	NA	2.4 (0.59–9.9)	-
	P-value		0.22	-
	HR (95% CI)	NC	2.6 (0.63–10.63)	-
	P-value		0.19	-
161	Larynx			
	Number	3	0	79
	HR_adj (95% CI)	2 (0.62–6.2)	NC	-
	P-value	0.25		-
	HR (95% CI)	3 (0.93–9.4)	NC	-
	P-value	0.065		-
208	Polycythaemia vera			
	Number	1	0	75
	HR_adj (95% CI)	0.73 (0.1–5.3)	NC	-
	P-value	0.76		-
	HR (95% CI)	1 (0.14–7.3)	NC	-
	P-value	0.99		-
142	Salivary gland			
	Number	1	0	58
	HR_adj (95% CI)	1 (0.14–7.3)	NC	-
	P-value	0.99		-
	HR (95% CI)	1.3 (0.18–9.3)	NC	-
	P-value	0.8		-
209	Myelofibrosis			
	Number	0	1	52
	HR_adj (95% CI)		1.7 (0.23–12.23)	-
	P-value	NC	0.6	-
	HR (95% CI)		2.5 (0.34–18.34)	-
	P-value	NC	0.37	-
160	Nose, nasal cavities, middle ear and accessory sinuses			
	Number	0	2	45
	HR_adj (95% CI)	NC	3.5 (0.85–15.85)	-
	P-value		0.082	-
	HR (95% CI)	NC	5.4 (1.3–22)	-
	P-value		0.02	-
140	Lip			
	Number	1	0	35
	HR_adj (95% CI)	1.5 (0.21–11.21)		-
	P-value	0.68	NC	-
	HR (95% CI)	2.2 (0.3–16)		-
	P-value	0.44	NC	-
206	Monocytic leukaemia			
	Number	1	0	16
	HR_adj (95% CI)	3.5 (0.46–26.46)	NC	-
	P-value	0.22		-
	HR (95% CI)	4.6 (0.6–35)	NC	-
	P-value	0.14		-
164	Mediastinum			
	Number	1	0	11
	HR_adj (95% CI)	6.5 (0.8–53)		-
	P-value	0.079	NC	-
	HR (95% CI)	5.7 (0.73–44.73)		-
	P-value	0.097	NC	-

*HR*_*adj*_ Adjusted hazards ratio, *HR* Unadjusted hazards ratio, *CI* Confidence interval, *IVF* Standard in vitro fertilization, *ICSI* Intracytoplasmic sperm injection, *ART* Assisted reproductive technology, *NC* Not calculated

### Sensitivity analyses: excluding cancer of unknown primary

No changes in risk-estimates or statistical significance were seen in sensitivity analyses, where diagnoses which could represent cancers of unknown primary were excluded from the definition of non-reproductive cancer.

## Discussion

In this study, we found that men who achieved paternity using ICSI or ART with donated spermatozoa had an increased risk of developing non-reproductive cancer. This risk was not present among men who conceived using standard IVF, which in Sweden is more commonly used in couples with female infertility [10]. Moreover, in a subgroup analysis, ICSI fathers were at an almost 2-fold and 4-fold increased risk of developing colorectal and thyroid cancer, respectively.

While such relationship between male infertility and cancer risk is yet to be fully understood, certain hypotheses have been put forward. With over 2300 genes (10% of the human genome) being involved in the reproductive function, it is plausable that certain genetic alterations impairing fertility could also promote oncogenesis [26, 27]. Genetic mutations which both impair male reproductive function and increase the susceptibility to develop malignancies, could partly explain the higher risk for colorectal cancer we observed in our study. Mutations in the mismatch repair genes MSH2 and MLH1 can lead to development of hereditary polyposis colorectal cancer (Lynch syndrome) [17, 19] as well as to oligozoospermia or non-obstructive azoospermia, respectively. ^18,20^. Our finding of an increased risk of thyroid cancer in men who fathered through ICSI treatment is in accordance with a previous study by Eiesenberg et al. ^13^. Although the underlying mechanism still is unclear, an underlying common genetic ground could also play a role here, as it has been shown that 1 st and 2nd degree-relatives of men with azoospermia have an increased risk of thyroid cancer [28]. Recent study from Valkna et al. ^29^ reported a nearly five-fold enrichment of pathogenic variants in hereditary cancer genes among infertile men compared to fertile controls (6.9% vs. 1.5%, *P* = 2.3 × 10⁻⁴). The implicated genes are either pleiotropic—associated with both human development and hereditary cancer (e.g., TSC1, PHOX2B, WT1, SPRED1, NF1, LZTR1, HOXB13)—or functionally involved in pathways essential for genomic stability, including mitosis, meiosis, and the repair of DNA double-strand breaks and interstrand crosslinks (e.g., monoallelic BRCA2 and biallelic FANCM). Given that colon cancer has a well-established genetic component, it is plausible that its occurrence may also be overrepresented in this context due to the shared genetic background. Interestingly, another analysis by Ramsay et al.^30^ showed relatives to oligozoospermic men to have higher risk for various cancers including colon cancer further confirming possible genetic background in the established associations in our analysis. Moreover, apart from genetic factors, lifestyle habits may also be linked to male subfertility and cancer, and thus could be a contributing factor for both disorders. Cigarette smoking, alcohol overconsumption and obesity have been found to be associated with poor semen quality [31–33], and with increased risk of several types of cancer [34–37], including colorectal cancer. Our analyses took paternal age and educational level into account, but unfortunately our register data did not contain information on lifestyle factors. However, regardless of the underlying aetiological and pathogenetic factors, men with impaired fertility seem to be at increased risk of not only reproductive but also non-reproductive cancers.

The growing body of evidence for a link between male infertility and an increased risk of non-reproductive cancer raises the question whether measures for long-term prevention of non-communicable diseases among subfertile men should be introduced. For young men seeking healthcare due to fertility issues, this might be one of few opportunities for otherwise relatively healthy men to also undergo a health check-up. Whether men with poor semen quality would benefit from cancer screening remains to be elucidated. An exemplary focus area would be to resolve whether the increased risk of cancer among infertile men is simply due to lifestyle factors, already targeted through other public health campaigns, or whether there are other underlying biological mechanisms. Nevertheless, elevated cancer risks in among relatively young men and first time fathers mandates further investigation and stratification to risk groups according to sperm counts and other sperm parameters.

A major strength of this study is the use of high-quality Swedish nationwide registers. The registers, being mandatory by law, have close to 100% coverage of the underlying population [38–42]. Some of the previously available data on this topic has relied on insurance-based databases [13], and therefore at risk of not being representative for the general population. As fertility treatment is included in the tax-funded healthcare system in Sweden, this further limits the selection bias of using ICSI treatment as a proxy for male infertility. Yet, achieved paternity through ICSI still has certain limitations. Men with most impaired reproductive function who failed to achieve paternity through ART were not included in the study. Moreover, despite controversial, in recent years ICSI treatment has been increasingly used in cases of non-male factor infertility. US data has shown increase in ICSI utilization from 36.4% in 1996 to 76.2% in 2012, with the largest relative increase among cycles without male factor infertility [43]. Despite this study clearly showing no benefit when it comes to fertilisation and pregnancy the question of ICSI for non-male factor infertility has risen worldwide [44]. Unfortunately, since we do not have data on semen quality we cannot state how much of the ICSI procedures are performed for non – male factor infertility. One factor which speaks in favor of that this might not be the case in Sweden is the fact that during the study period the majority of the IVF procedures in Sweden are performed in nonprivate clinics with public funding. If men with the most pronounced impairment of spermatogenesis are those at highest risk of future malignancies, both mentioned potential selection biases would tend to act in favor of the null hypothesis and can, therefore, not explain the statistically significant findings reported by us. Finally, ICSI and conception following use of donated spermatozoa, were both considered as proxy of poor male fertility. However, donated male gametes are also used in cases of severe genetic disease in the male partner, aiming to prevent transmission to the offspring. Since only two of the 192 non-reproductive cancer seen in the ICSI/donated spermatozoa group, manifest serious genetic disease can hardly explain the increased cancer risk in those men. The lack of information on paternal smoking habits can be regarded by some as a major limitation due to known cancerogenic effect. However, to the best of our knowledge there are no studies showing men who undergo fertility treatment to have elevated consumption of tobacco products. Another limitation is the lack of information on several other potential confounders, including body mass index, medication use, diabetes, and other clinical or lifestyle factors that may be associated with both infertility and cancer risk. This limitation is inherent to population-based registry studies, which typically rely on routinely collected administrative data and therefore lack detailed individual-level information on such variables. Consequently, residual confounding cannot be fully excluded and should be considered when interpreting the observed associations. Another issue which necessitates caution is the fact that some of the cancer forms are represented by relatively small numbers as is the case for thyroid cancer.

## Conclusion

In conclusion, in this large registry-based cohort, we found that men who achieved paternity using ICSI were at an increased risk of certain types of non-genital cancers such as colorectal and thyroid cancer. These results represent an additional argument for considering initiation of disease preventing measures in men seeking healthcare due to fertility problems. Thus, in countries where colorectal cancer screening has already been introduced, lowering the age at which the screening should be initiated in men with severe infertility, could be considered. Since scrotal ultrasound is already a part of andrological examination, adding thyroid ultrasound scanning to the standard andrological work up, appears as a feasible option.

## Supplementary Information

Below is the link to the electronic supplementary material.

Supplementary Material 1
