# Supplementary material for: Risk of non-reproductive cancer in men from couples conceiving through assisted reproduction: a Swedish nation-based register study
Source: Eur J Epidemiol. 2026 Feb 21;41(4):495–505. doi: 10.1007/s10654-026-01368-4 (PMC13331865; doi:10.1007/s10654-026-01368-4)
Supplement: Supplementary file 1 — Supplementary Material 1 [file 10654_2026_1368_MOESM1_ESM.docx]

Dear Members of the Editorial Team,

We thank you and the reviewers for your valuable feedback on our manuscript. We appreciate the time and effort that went into first and second review process and the thoughtful suggestions that were provided.

In response to the comment of the Editor, we have made minor revision to the manuscript. Our detailed response is included below.

We hope that the revised version meets the expectations of the journal and that the manuscript is now suitable for publication.

Thank you again for the opportunity to revise and resubmit our work.

Sincerely,
Angel Elenkov MD, PhD

Lund University
angel.elenkov@med.lu.se

Editor: Table 1 in its current form is uninformative. The layout should be revised so that the columns represent the exposure categories and the rows list potential confounders. Presenting data only for age and education is insufficient. Additional variables that may confound the association—such as BMI, medication use, diabetes, and other relevant factors linked to both infertility and cancer risk—should also be included.

**Reply:** We agree with the Editor that a table presenting only age and educational level is of limited informativeness. Unfortunately, the additional variables suggested—such as BMI, medication use, diabetes, and other potential confounders—are not available in our dataset. This limitation is inherent to population-based registry studies and has been explicitly acknowledged as a major limitation in the Discussion (lines 294-300). In light of this, and to avoid presenting an uninformative table, we have removed Table 1 and instead incorporated the available descriptive information into the Results section (lines 170 - 176).
